# Review on the Parameters of Recycling NdFeB Magnets via a Hydrogenation Process

**DOI:** 10.1021/acsomega.3c00299

**Published:** 2023-05-08

**Authors:** Alireza Habibzadeh, Mehmet Ali Kucuker, Mertol Gökelma

**Affiliations:** † Department of Environmental Engineering, Izmir Institute of Technology, 35430 Izmir, Türkiye; ‡ Department of Materials Science and Engineering, Izmir Institute of Technology, 35430 Izmir, Türkiye

## Abstract

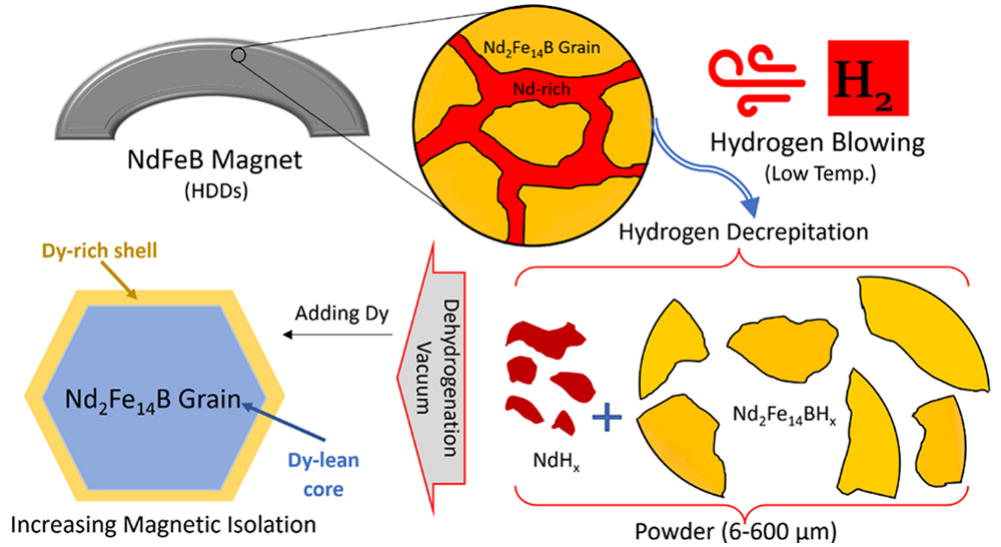

Regarding the restrictions recently imposed by China on the export of rare-earth elements (REEs), the world may face a serious challenge in supplying some REEs such as neodymium and dysprosium soon. Recycling secondary sources is strongly recommended to mitigate the supply risk of REEs. Hydrogen processing of magnetic scrap (HPMS) as one of the best approaches for magnet-to-magnet recycling is thoroughly reviewed in this study in terms of parameters and properties. The processes of hydrogen decrepitation (HD) and hydrogenation–disproportionation–desorption–recombination (HDDR) are two common methods for HPMS. Employing a hydrogenation process can shorten the production route of new magnets from the discarded magnets compared to other recycling routes such as the hydrometallurgical route. However, determining the optimal pressure and temperature for the process is challenging due to the sensitivity to the initial chemical composition and the interaction of temperature and pressure. Pressure, temperature, initial chemical composition, gas flow rate, particle size distribution, grain size, and oxygen content are the effective parameters for the final magnetic properties. All these influencing parameters are discussed in detail in this review. The recovery rate of magnetic properties has been the concern of most research in this field and can be achieved up to 90% by employing a low hydrogenation temperature and pressure and using additives such as REE hydrides after hydrogenation and before sintering.

## 1 Introduction

The third group of elements in the periodic table, which includes scandium, yttrium, and lanthanides and is referred to as rare-earth elements (REEs), has recently become a subject of concern due to the risk of supply in the near future.^1^ The demand for rare-earth elements has been steadily increasing due to their essential role in clean energy generation and a green economy.^2^ REEs are used in various applications such as catalysts, permanent magnets, ceramics, glass, batteries, etc. and various industries such as electronics, automotive, and renewable energy.^3^ The European Commission considers REEs as the most critical group of raw materials with the highest supply risk.^4^ The US Department of Energy (DOE) has also declared the five most critical REEs: neodymium (Nd), europium (Eu), terbium (Tb), dysprosium (Dy), and yttrium (Y).^5^ Since the early 1990s, the main global supply (more than 90%) of REEs came from China. Due to increasing domestic demand in recent years, China has restricted exports of these elements.^6^

The permanent magnet and catalyst industries, which consume 23% and 24% of all REEs, respectively, were the main consumers of REEs.^7^ The permanent magnet industry relies on only one type of permanent magnet: namely, the neodymium–iron–boron (NdFeB) magnet. Consequently, the consumption of Nd, Dy, Pr, Gd, and Tb to produce this type of magnet is considered the main application of REEs.^3^ According to various projections, the world will face serious difficulties in the supply of some REEs such as Dy, Nd, and Tb in the upcoming decades.^8,9^ Filippas et al. (2020)^10^ predict an increase in Nd and Dy demand during 2017–2030 based on existing permanent magnet technology, including electric vehicles (EVs), hybrid electric vehicles (HEVs), electric bicycles, wind turbines, and robotics, with an overall increase in Nd and Dy supply demand of 191% and 168%, respectively. These challenges led researchers to look for alternative solutions to provide these important and strategic elements. For this reason, various types of research have been carried out to recover these elements from secondary sources such as end-of-life (EoL) NdFeB magnets.^11,12^

**Figure 1:**
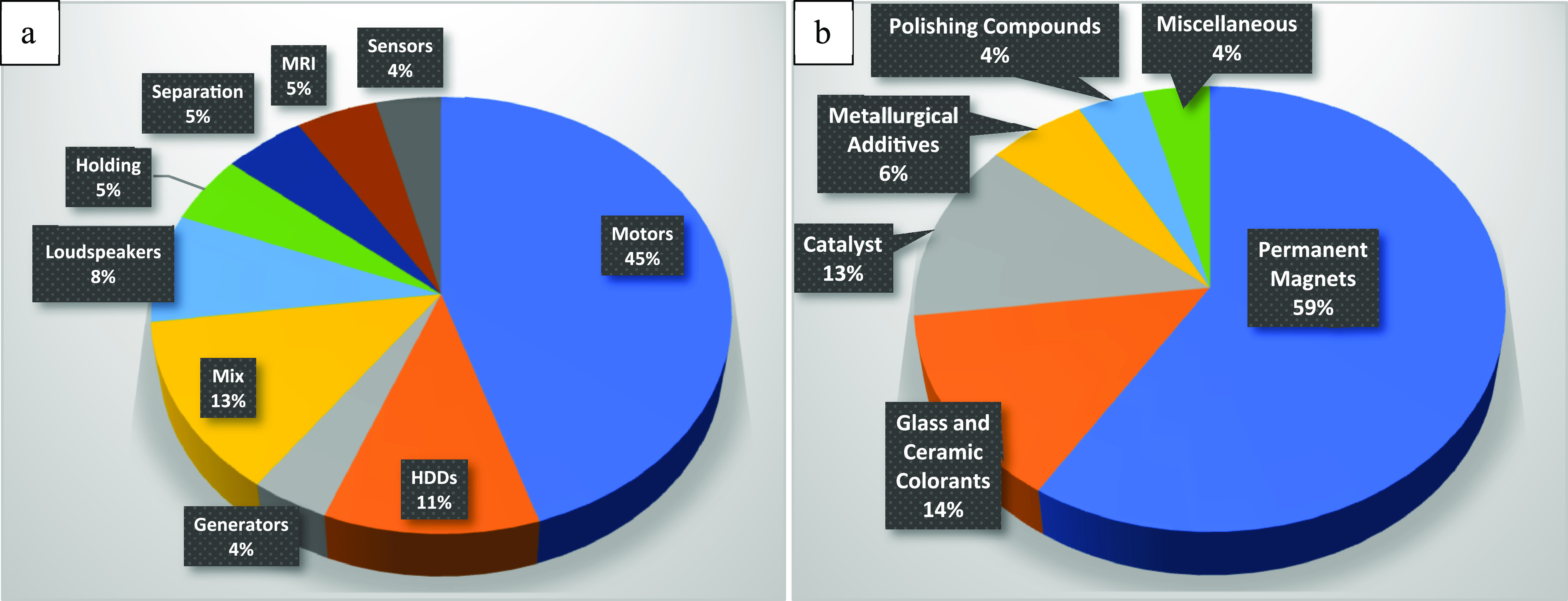
(a) Application of permanent magnets by market ($) 2019. Adapted from ref (14). (b) Distribution of neodymium consumption (%), reported by the U.S. Geological Survey (USGS) in 2020.

The NdFeB magnets were introduced in the 1980s and became the first choice for a variety of applications. Due to the optimal power-to-size ratio, these magnets are widely used today; from small devices such as hard disk drives (HDDs) of PCs and laptops, smartphones, and music players to large industrial applications such as EVs and HEVs, wind turbines, refrigerators, and MRI devices as shown in Figure 1.^13−15^ Depending on the application, these magnets have different weights and life cycles. The magnets used in electronic devices weighing 1–30 g reach EoL after 2–5 years, those of EVs and HEVs weighing more than 1 kg reach EoL after 16 years, and the oversized NdFeB magnet used in generators of modern wind turbines weighing 1000–2000 kg works for 20–30 years.^10,16^

Since there are no working commercial processes for recycling REEs, buying these magnets appears to be more economical than reprocessing complex scrap magnets.^17^ To date, most REE recycling activities are still in the research and development stage, and no large-scale industrial process has been established, while it is estimated that recycled REEs will be the main raw material supplier for magnet production in 2030.^18^ Moreover, the existing recycling practice mainly focused on the EoL magnets in HDDs due to their accessibility and availability. In the next decade, on the other hand, most scrap magnets will come from the energy and automotive sectors, including wind turbines, EVs, and HEVs.^19^

Over the years, various methods for recycling EoL NdFeB magnets have been introduced, which can be generally categorized as hydrometallurgical methods, including leaching and bioleaching, and pyrometallurgical methods, including recasting and melt spinning, high-temperature processing, and hydrogen processing of magnetic scrap (HPMS).^20^ Most research in the field of recycling EoL permanent magnets has focused on hydrometallurgical processes to recover neodymium oxides followed by the energy-intensive step of molten salt electrolysis to obtain elemental Nd.^21,22^ Recently, a biohydrometallurgical method based on biosorption has been developed to reduce the environmental footprint of hydrometallurgy, which produces a large amount of liquid waste.^23,24^ In general, all recycling methods for processing EoL NdFeB magnets are complex, but each method has some advantages and disadvantages. For instance, in the recasting and melt spinning method, the power consumption is high and 20–30% of the magnet is lost (low efficiency), while the oxygen content is low.^20^ The hydrometallurgical routes are very complex with a high environmental footprint due to the huge amount of liquid waste generated during the process. At the same time, the efficiency is much higher (typically above 90% and can reach 98% by an acid-baking process with nitric acid) than that of the pyrometallurgical route.^25^ The environmental footprint of pyrometallurgical routes is even higher than that of the hydrometallurgical route, since some of them include a liquid metal extraction step except for recasting and melt spinning.^26^ The recently developed bioleaching method significantly reduces the cost and environmental footprint, while achieving 100% efficiency of Nd leaching takes 14 days, which is not economically feasible for industry.^27^ Among all attempts to recycle NdFeB magnets, the magnet-to-magnet recycling method with high efficiency (∼90%) is the shortest route to produce new magnets from EoL permanent magnets through the hydrogenation process when the waste magnet is clean and is not oxidized.^20,28^ This study reviews all the research that has been carried out in the field of recycling EoL permanent magnets via the hydrogenation process and presents the variable parameters and influencing factors.

## 2 NdFeB Magnets

**Figure 2:**
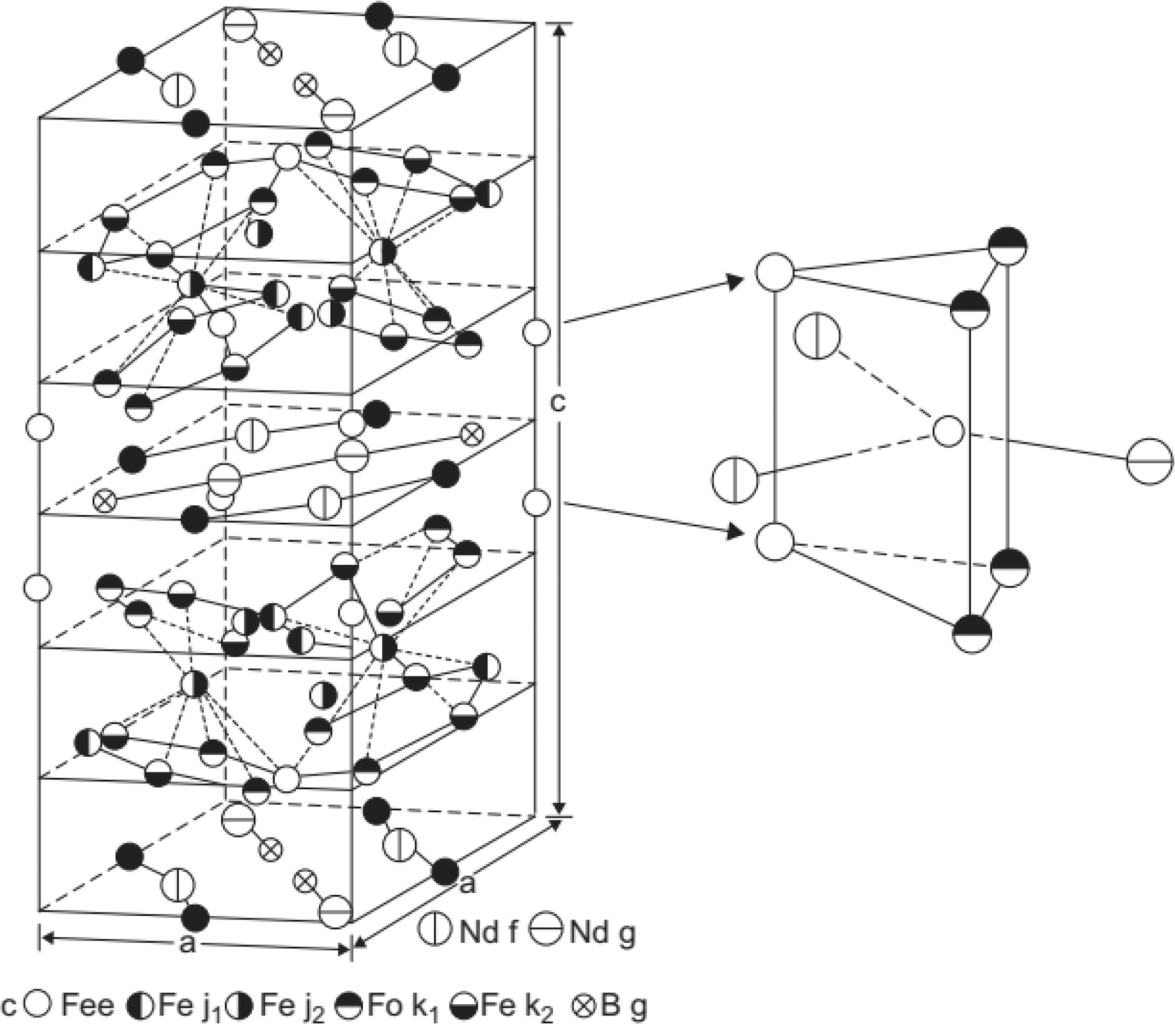
Tetragonal crystal structure of Nd_2_Fe_14_B. The *c*/*a* ratio has been changed so that puckered hexagonal nets can be more clearly seen. The magnified region is trigonal prisms that include the boron atoms. Reprinted with permission from ref (29). Copyright 1984 APS.

The intermetallic compound Nd_2_Fe_14_B with a tetragonal structure (Figure 2)^29^ opened a new era of permanent magnets in terms of maximum energy product (BH)_max_, ending the era of extensive use of samarium–cobalt magnets.^30^ Samarium–cobalt magnets (SmCo_5_) were introduced in the mid-1960s. They brought a revolution in the field of magnets, providing a maximum energy product of 240 kJ/m^3^, 3 times higher than the previous technology (Alnico magnets). However, due to the rarity and high price of samarium, unreliable sources, and fluctuating cobalt price, an alternative to SmCo permanent magnets was needed.^31^ Therefore, NdFeB permanent magnets, which can reach over 400 kJ/m^3^ for (*BH*)_max_, became the only and best choice for many industrial applications. The discovery of a neodymium-based magnet was announced simultaneously by two different companies in Japan and the USA in 1984, with one small difference. Sumitomo Corporation introduced the NdFeB permanent magnet in Japan, which has a higher Nd chemical composition than that of General Motors in the US. This additional amount of Nd results in the formation of an Nd-rich phase (layer) surrounding the grains, causing magnetic insulation of the grains and enhancement of magnetic properties. In addition, the high value of (*BH*)_max_ in NdFeB magnets is also attributed to the microstructure of the intergranular phases which can be modified by adding some heavy rare earth elements (HREEs) such as Dy and Tb.^32^

### 2.1 Magnetic Properties

**Figure 3:**
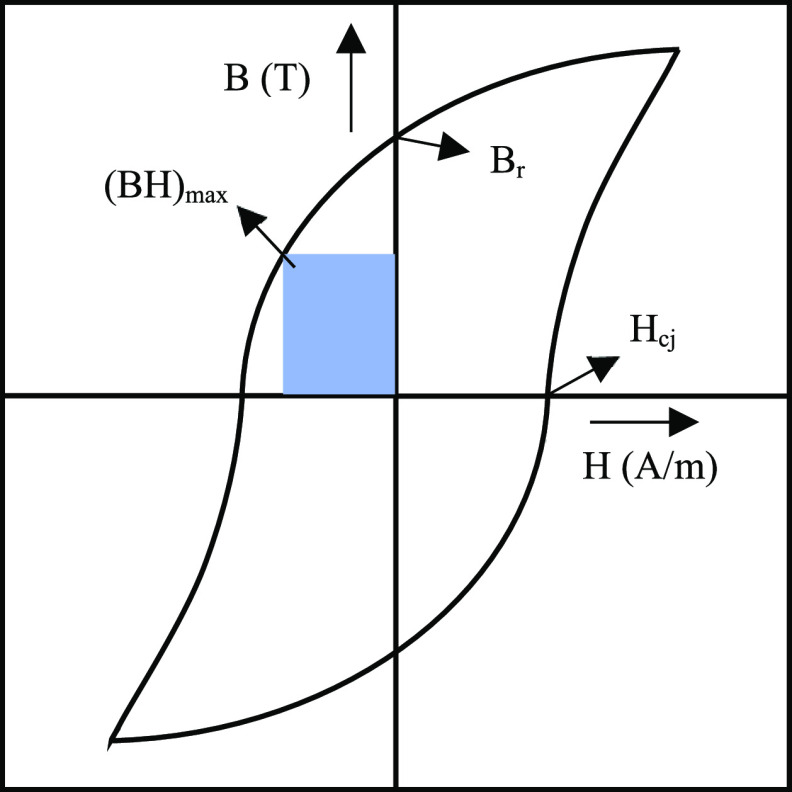
Typical intrinsic hysteresis loop of a permanent magnet, representing the remanence *B*_r_, coercivity *H*_cj_, and maximum energy product (*BH*)_max_. Adapted with permission from ref (35). Copyright 2009 Elsevier.

Magnetic materials include a wide range of materials required for the development of environmentally friendly technologies, which can be broadly divided into hard and soft magnets. Hard magnetic materials, known as permanent magnets, are more resistant to demagnetization. The normal magnetic properties (preferred by engineers) are defined by the relationship between the magnetic field strength (*H*) in the magnet and the net magnetic flux density (*B*).^33^ The more common hysteresis loop, which is the energy required to magnetize and demagnetize the magnets, is defined based on the intrinsic properties of the magnetic materials. The intrinsic properties preferred by material scientists include remanence (*B*_r_) and coercivity (*H*_cj_). Remanence represents the magnetic flux density when the magnetic field strength is zero and is expressed in units of tesla or gauss, and coercivity represents the magnetizing field strength when the magnetic flux density is zero and is expressed in ampere-turn/m or oersted (Oe). According to the definition of remanence and coercivity, the intrinsic hysteresis loop can be drawn as shown in Figure 3. The maximum energy product (*BH*)_max_, which represents the power of the magnet, is the product of the multiplication of remanence and coercivity and is graphically the largest rectangle that can be fitted into the second quadrant of the intrinsic hysteresis loop, as shown in Figure 3.^31^ In other words, (*BH*)_max_ (given in kJ/m^3^ or MGOe) represents the energy required to magnetize and demagnetize the magnet.^34^

### 2.2 Primary Production of NdFeB Magnets

**Figure 4:**
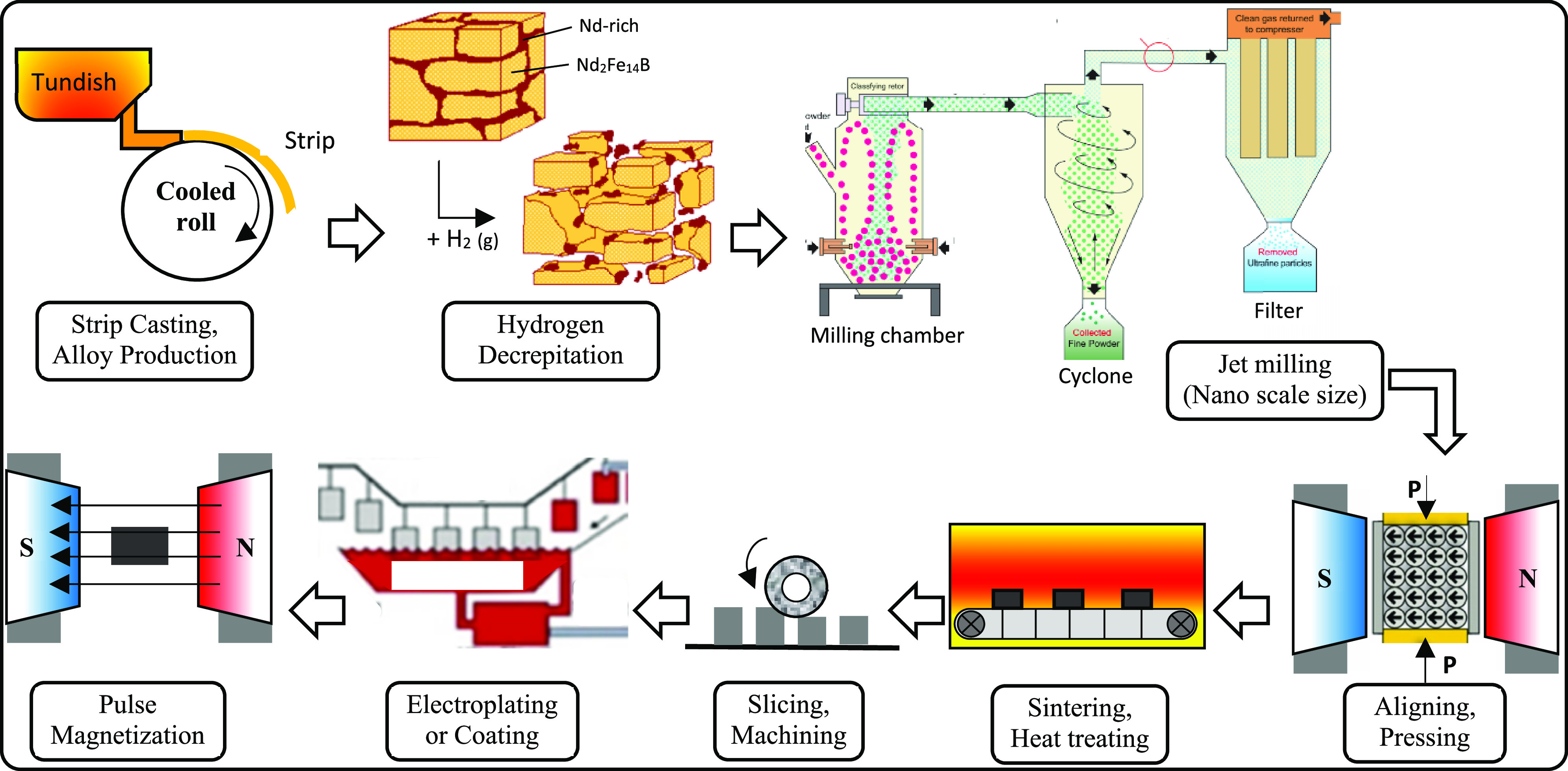
Primary production process steps for NdFeB magnets. Adapted from ref (14).

NdFeB magnets are divided into two categories: *sintered* magnets and *resin-bonded* magnets, each manufactured differently. Rapid solidification or melt spinning is used to produce isotropic bonded magnets and anisotropic hot-deformed NdFeB magnets. The sintered neodymium magnets, which make up the majority of the production, are manufactured using the same process that was used to produce SmCo magnets.^30^ This process begins with a strip casting of individual Nd, Fe, and B ingots to produce an alloy ingot, which is then decrepitated with hydrogen to produce a powder with a particle size in the micrometer range. Further comminution down to the nanoscale is accomplished by jet milling, to increase pressing efficiency. After pressing in a magnetic field, the samples are sintered and subjected to heat treatment, followed by machining, coating, and magnetization in the final stage, as shown in Figure 4.^14^

### 2.3 The Necessity of Recycling NdFeB Magnets

**Figure 5:**
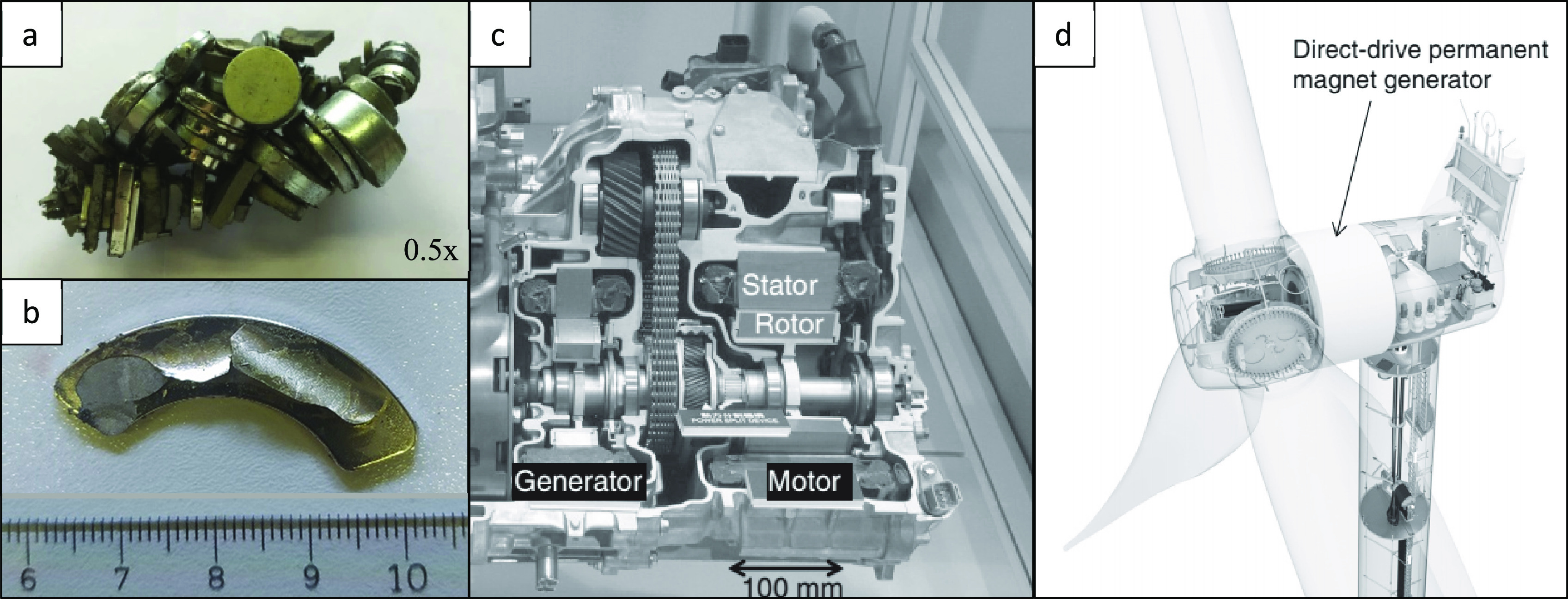
Different applications of NdFeB magnets. (a) Audio system. Photograph courtesy of Alexandru Lixandru. Reprinted with permission from ref (40). Copyright 2017 Elsevier. (b) HDD, (c) generator and motor in a hybrid automobile and (d) wind turbines. Photograph courtesy of I. R. Harris. Reprinted with permissions from ref (41). Copyright 2012 Elsevier.

Although the NdFeB magnet is called a permanent magnet, it is not truly permanent, but it has the longest life span compared to other magnets. At present, the maximum lifetime of NdFeB magnets in the industry can reach 30 years in wind turbines.^16^ However, the actual life span of NdFeB magnets can vary significantly depending on the use and operating conditions. A high operating temperature (low Curie temperature of NdFeB magnet; *T*_c_ = 310 °C), corrosive environment, and demagnetization field can shorten the life of NdFeB magnets.^36,37^ Generally, most electronic devices that use NdFeB magnets are disposed of before the magnet loses its power, such as HDDs (2–5 years).^38^ Through the systematic collection of e-waste, the discarded magnets could have sufficient magnetic strength for reuse. Still, due to the continuous development of technology, the geometric shape of the magnet is not the same as that of newly developed magnets. Thus, the process of reshaping the magnet means reproducing or recycling the magnet. Some of the most common applications of NdFeB magnets, including audio systems, HDDs, electric motors, and wind turbines, are shown in Figure 5.

Since most applications are for sintered NdFeB magnets, recycling practices are also focused on sintered NdFeB magnets rather than resin-bonded NdFeB magnets. Due to the high strength and very compact structure of sintered NdFeB magnets, hydrogen decrepitation is a necessary step to break down the magnet mass. On the other hand, the resin-bonded NdFeB magnets have very low strength and can be easily crushed by heating and removing the resin and epoxy used in their fabrication.^39^

## 3 Hydrogen Processing of Magnetic Scrap (HPMS)

Efforts have been made to introduce technical and technological innovations in recycling EoL NdFeB magnets (mainly HDDs), focusing on magnet-to-magnet recycling by hydrogenation. The advantage of this approach is not only the reduction of supply risk but also the saving of 45% of energy consumption compared to primary magnet production from ores, making this approach more economical by reducing production costs by 53% and more environmentally friendly by reducing CO_2_ emissions by more than 11 tons per ton of magnets recycled.^21,42^ The magnet-to-magnet recycling process for HDDs consists of two distinct stages: first, the collection and conversion of the EoL magnets to powder, and second, the production of new NdFeB magnets. In the first stage, after collection, the discarded HDDs are automatically or manually disassembled to remove the magnet assembly (MA),^43^ which can then be demagnetized and decoated before the hydrogenation process. The second step is to homogenize the EoL NdFeB powder with 5% new rare-earth material in the hydrogen mixing reactor, and then the mixed powder is milled and homogenized again. Finally, the products are sintered and magnetized in block form.^44^ The magnetic properties and performance of these blocks are equivalent to or better than those of new blocks obtained from ores, and the recovery rate is more than 90%.^45^

Hydrogen processing of magnetic scrap (HPMS) as one of the most efficient approaches for magnet-to-magnet recycling has been developed over the years. Still, it has not been able to attract the attention of recyclers and replace the current separation steps. In this review, two different HPMS are explained in detail. The first is the hydrogen decrepitation (HD) process, which is already used in the primary production of NdFeB magnets to convert the alloy into powder form before further comminution.^46^ The second process, named HDDR, which consists of two different steps, namely hydrogenation–disproportionation (HD) and desorption–recombination (DR), was recently developed to produce ultrafine and uniform grains.^33^

## 4 Hydrogen Processing Mechanism and Thermodynamics

When the magnets are exposed to hydrogen gas, the hydrogenation process is initiated at the Nd-rich phase and triple points.^47^ The HPMS is based on the difference in reactivity of the Nd-rich phase and the matrix of Nd_2_Fe_14_B grains upon exposure to hydrogen gas. Due to the formation of neodymium hydride in the Nd-rich phase and induced expansion, the entire structure of the magnet is collapsed and converted into powder form.^48,49^ Due to some differences between the two existing hydrogenation methods (HD and HDDR) and the similar terminology, it is necessary to present them separately.

### 4.1 Hydrogen Decrepitation and Dehydrogenation Process

Hydrogen, the smallest atom, is very reactive and easily penetrates the grain boundaries of many metals. In practice, the daily life of a metallurgist was ended by preventing hydrogen from penetrating into the metal to avoid brittleness and brittle fracture. The mechanism of this type of failure is that hydrogen easily diffuses into the grain boundaries and creates pressure at the weakest point, which leads to microcracks that begin to propagate in the grain structure. As a result of this propagation, the specimen experiences inelastic strain, which significantly decreases the fracture strength.^50^ This property of hydrogen forms the basis of the targeted hydrogenation process for the decomposition and decrepitation of a wide range of materials.

The process of hydrogen decrepitation (HD), which is used in the primary production and recycling of Nd magnets, takes place at low temperatures, even at room temperature, so that the entire microstructure decomposes only in powder form and the grain size is reduced. The operating temperature of the HD process is 25–400 °C, resulting in particle sizes in the range of 6–600 μm after hydrogenation.^51^ The most important advantage of the HD process compared to previously used processes such as mechanical crushing is the production of a very friable and demagnetized hydrogenated powder prior to jet milling.^33^

**Figure 6:**
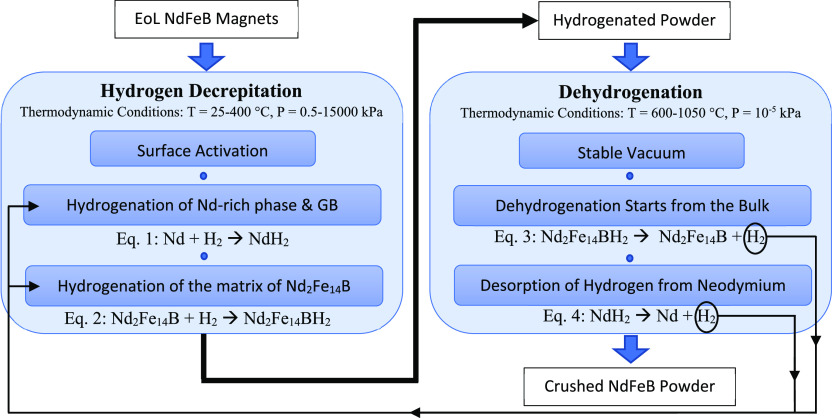
Mechanism flow sheet for recycling EoL NdFeB magnet through hydrogen decrepitation and dehydrogenation.

**Figure 7:**
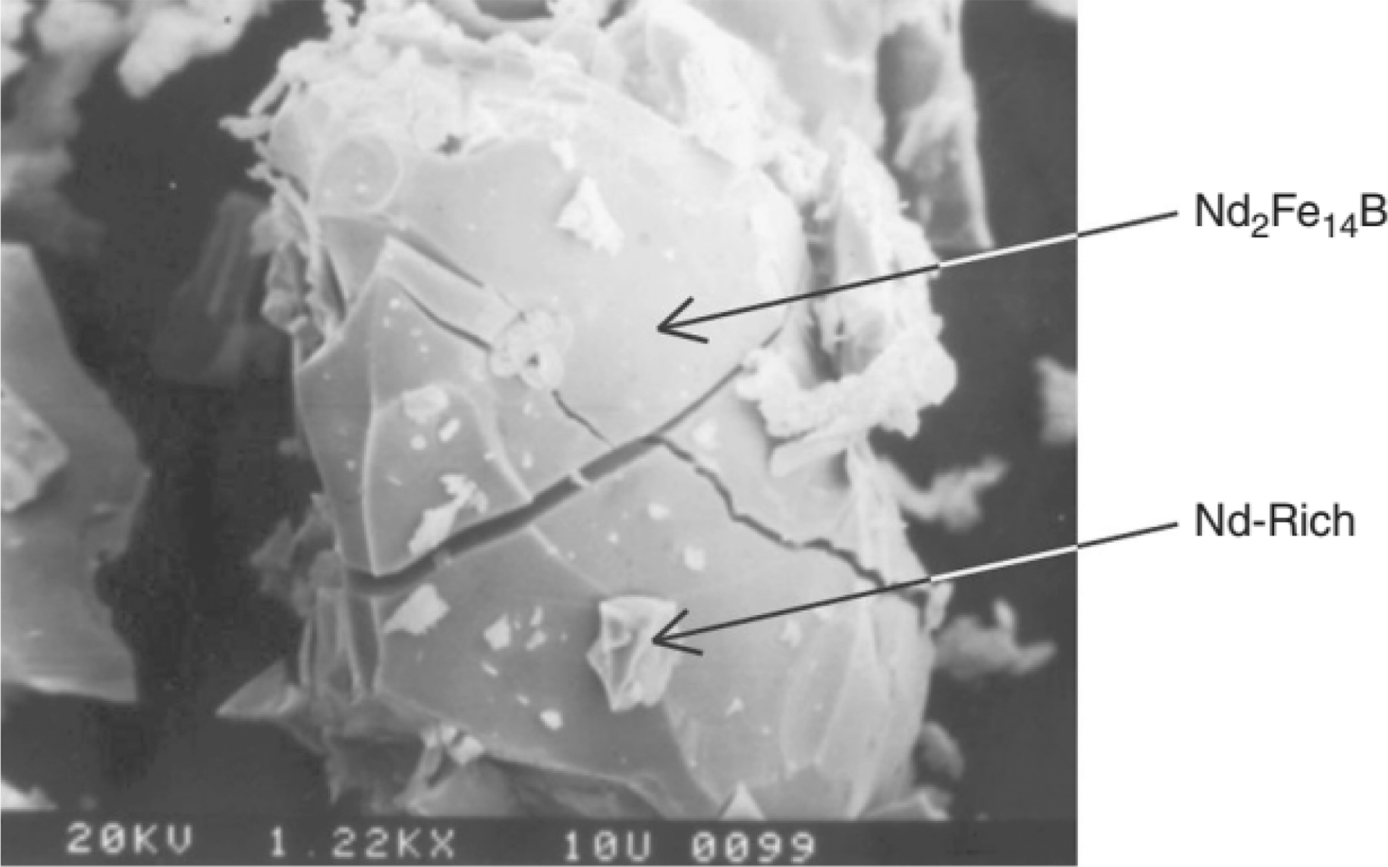
A single grain of Nd_2_Fe_14_B after the hydrogenation process. The trans-granular crack is shown in the bulk (1220×). Reprinted with permission from ref (33). Copyright 2012 Elsevier.

As shown in Figure 6, the HD process starts with the surface activation of the scrap magnet, when it has a coating, such as in the magnet assembly in HDDs. Then, the Nd-rich region expands by diffusion of hydrogen and the formation of neodymium hydride, which causes cracks in the entire structure.^51^ More exposure to the hydrogen gas completes the hydrogenation of the bulk until no further absorption of hydrogen is observed. The change in volume (Δ*V*) of the grain boundary is 3 times the change in the grains, resulting in a strain that decrepitates the magnets.^52^ As can be seen in a single Nd_2_Fe_14_B grain in Figure 7, a trans-granular crack confirms the brittleness of the hydrogenation product. This characteristic affects jet milling, which is the next step of magnet production, and increasing its efficiency can reduce the production cost by 25%.^33^

The stoichiometric coefficients of the reactions were simplified in Figure 6, and they can vary depending on the temperature and pressure of the hydrogenation process.^40,52−54^ For example, the stoichiometric coefficient of H_2_ might be higher. There are three neodymium hydrides, the most stable of which is NdH_2_, but under certain circumstances, such as high H_2_ concentration, other neodymium hydrides also occur, including Nd_2_H_5_ and NdH_3_.^55,56^ The presence of NdH_3_ after hydrogenation has also been confirmed by Michalski et al.^52^ Since NdH_3_ forms at a much higher pressure than NdH_2_, the possibility of detecting neodymium hydride with an index greater than two decreases with decreasing hydrogenation pressure.^57^ Therefore, the more accurate form of eqs 1 and 2 in Figure 6 is as follows: *x* and *y* depend on pressure and temperature.^51^
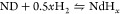
5
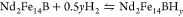
6

**Figure 8:**
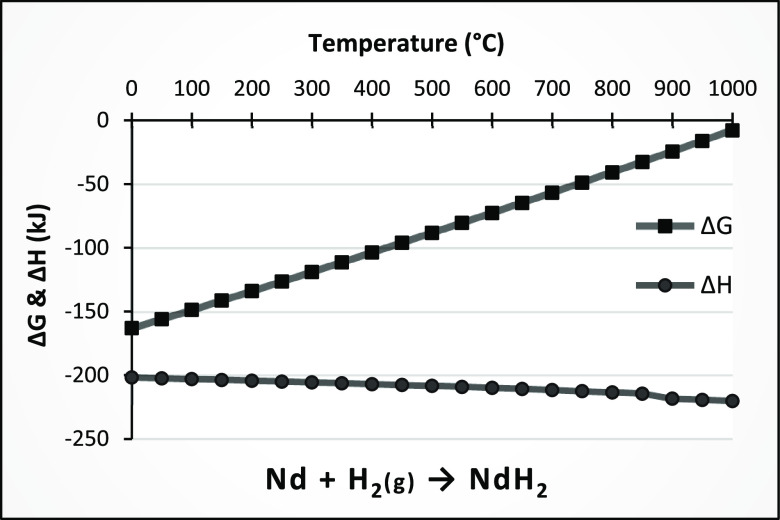
Gibbs free energy and enthalpy of formation of neodymium(II) hydride. Extracted from HSC software.

Based on the Gibbs free energy of NdH_2_ (eq 1), as shown in Figure 8, the formation of NdH_2_ is more likely at lower temperatures than at higher temperatures. Despite eq 1, there is no thermodynamic information about eq 2, Nd_2_H_5_, and NdH_3_ in the HSC and Factsage software, so it can only be said that the neodymium hydride is more stable at lower temperatures and no further thermodynamic evaluation of complete hydrogenation can be made. The insufficient thermodynamic information on these hydrides may be the subject of future work to determine the values of thermodynamic functions, including Δ*G*, Δ*H*, and Δ*S*.

**Figure 9:**
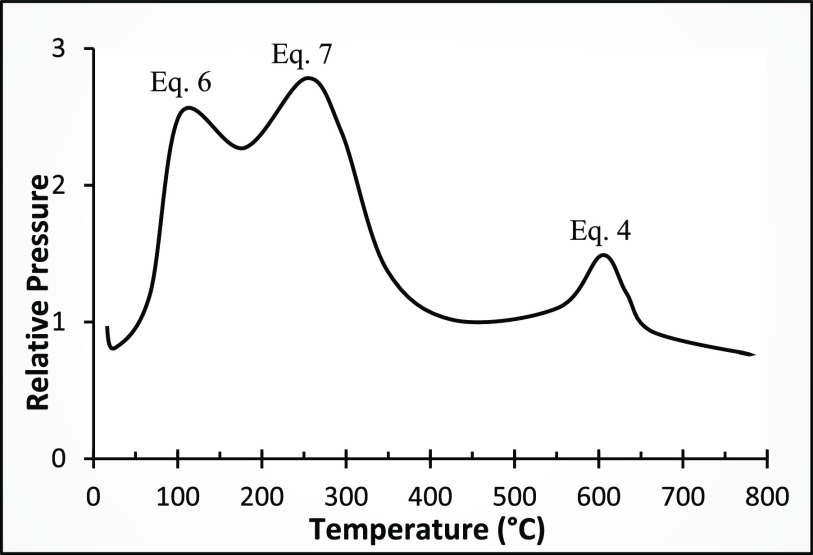
Three stages of dehydrogenation mechanism by increasing temperature. (Stage 1, eq 6): Nd_2_Fe_14_BH_*y*_ → Nd_2_Fe_14_B + 0.5*y*H_2_. (Stage 2, eq 7): NdH_2.7_ → NdH_2_ + 0.35H_2_. (Stage 3, eq 4): NdH_2_ → Nd + H_2_. Adapted with permission from ref (53). Copyright 2008 Elsevier.

As shown in Figure 6, the hydrogen should be removed during the process called dehydrogenation after the production of hydrogenated powder. By designing a closed-loop apparatus the removed hydrogen gas can be reused. Based on the zeroth thermodynamic law, by reducing the pressure to 10^–5^ kPa (or mbar) at elevated temperature (up to 1000 °C), the hydrogen in the structure of neodymium hydride is forced to fill the vacuum by reproducing H_2_ gas, leaving behind a highly reactive NdFeB powder ready for jet milling, pressing, sintering, and further processing to produce new NdFeB permanent magnets.^53^ The H_2_ gas is continuously pumped out during dehydrogenation until the vacuum becomes stable, which means that no further dehydrogenation is possible under these specific thermodynamic conditions or all hydrogen has been removed from the system.^58^ The dehydrogenation as shown in Figure 6 begins with the removal of hydrogen from the Nd_2_Fe_14_BH_*x*_ grains. Then it continues with the removal of hydrogen from the neodymium hydrides due to its stability. This mechanism is based on the presence of neodymium(II) hydride, while Zakotnik et al.^53^ considered three stages of dehydrogenation in the presence of NdH_2.7_, as shown in Figure 9. They reported that during heating under vacuum at a rate of 5 °C/min, after dehydrogenation of the Nd_2_Fe_14_BH_*x*_ grains, the NdH_2.7_ decomposes first to NdH_2_ and in the last stage to Nd according to eqs 6, 7, and 4, respectively. The experiments performed by Önal et al.^59^ also show that neodymium hydride with a hydrogen index higher than 2 decomposes to NdH_2_ in the second stage of dehydrogenation and Nd in the third stage via reaction 8.
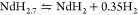
7
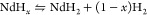
8

Based on the Δ*H* diagram (Figure 8), the formation of neodymium hydride is an exothermic reaction, which means that the reverse direction to dehydrogenation is endothermic, confirming the data presented by Zakotnik.^58^ Therefore, the minimum temperature for complete dehydrogenation is 600 °C.^60^ Moreover, Li et al.^48^ showed that the hydrogen content of the powder after hydrogenation depends on the hydrogenation temperature. They studied different temperatures in the range of 550–800 °C and demonstrated that 650 °C ensures the highest possible degree of dehydrogenation and the hydrogen content is 66 ppm and does not decrease further when the temperature is increased above 650 °C.

Although some efforts to recycle EoL NdFeB magnets have performed hydrogenation and dehydrogenation sequentially, this order is not necessary. Dehydrogenation can be performed during other subsequent high-temperature steps, such as sintering. For example, in the primary production of NdFeB magnets, dehydrogenation occurs during vacuum sintering at 1080 °C for 1 h.^44^ The other example is from Harris,^33^ the pioneer in developing the hydrogenation process for recycling neodymium magnets, where he and his colleagues proposed partial degassing (PD) after hydrogenation to improve the final magnetic properties. This is followed by fully degassing (FD) during vacuum sintering.

### 4.2 Hydrogenation–Disproportionation–Desorption–Recombination (HDDR) Process

**Figure 10:**
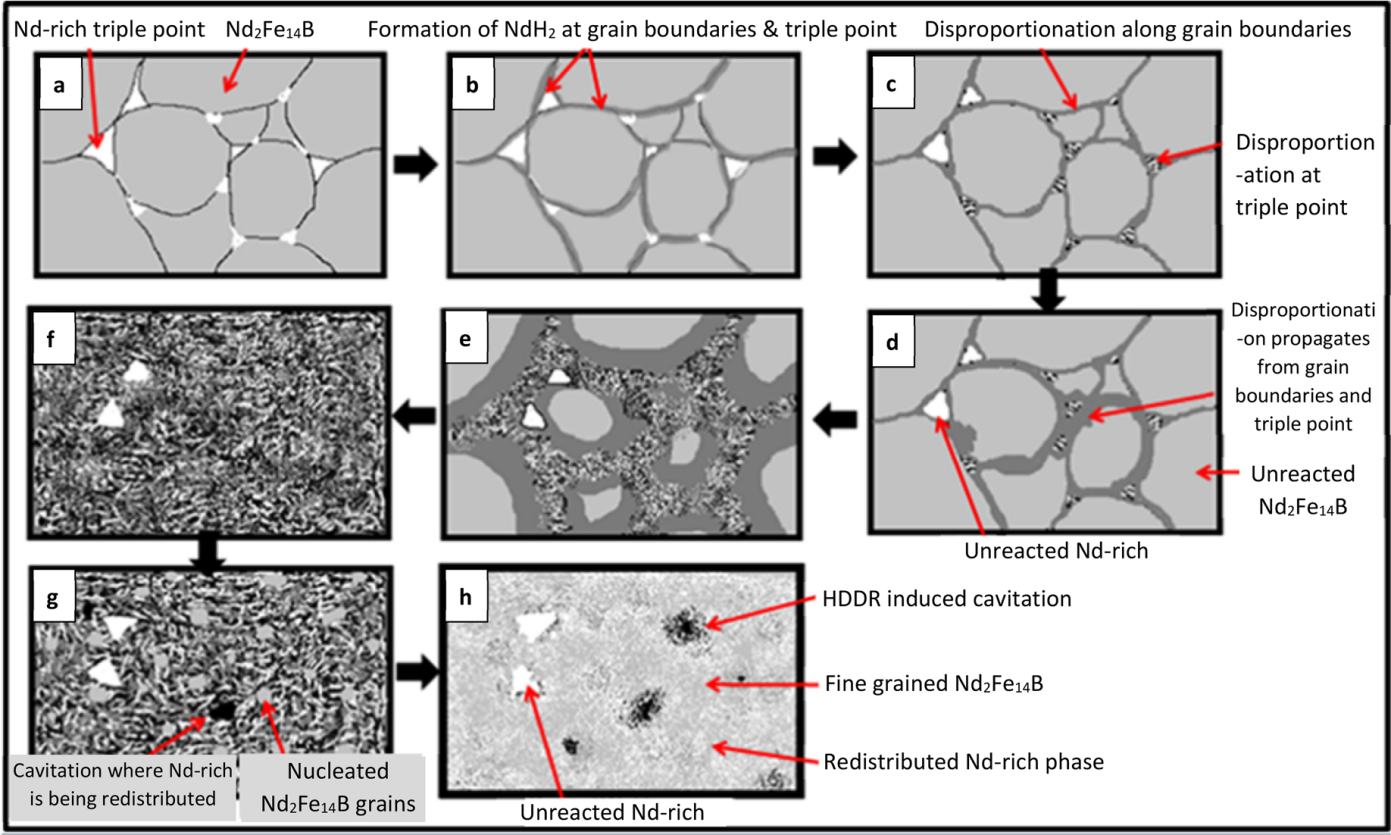
Representation of the HDDR microstructural evolution: (a) microstructure of NdFeB magnet before HDDR process; (b) initiation of hydrogenation; (c) initiation of disproportionation; (d, e) propagation of disproportionation; (f) fully disproportionated microstructure; (g) desorption and recombination; (h) redistributed microstructure. Reprinted with permission from ref (47). Copyright 2016 Elsevier.

The HDDR process was introduced by Mitsubishi Materials in Japan in 1990 and was developed to produce ultrafine grains. The main difference between the HDDR and HD processes is the higher operating temperature (750–950 °C) of hydrogenation in the HDDR process. The experimental evidence confirmed that the hydrogenation performed at higher temperatures in the HDDR process leads to more refined powder than the HD process with a grain size of 0.3 μm.^30^ Moreover, due to hydrogenation at elevated temperatures (at least 650 °C), the intermetallic compound Nd_2_Fe_14_B disproportionated to its constituents, including α-Fe, Nd, and Fe_2_B.^60^ The microstructural changes in each phase of the HDDR process have been studied by Sheridan et al.,^47^ showing the transformation of the phases during processing, as in Figure 10.

In the pure hydrogen atmosphere, hydrogenation begins with introducing hydrogen gas into the magnet (Figure 10a). As a result, the neodymium hydride forms at the grain boundaries and triple point (Figure 10b). By increasing the temperature, disproportionation is also initiated in the same areas (Figure 10c) and then propagates to the grains (Figure 10d,e) until the entire structure of Nd_2_Fe_14_B transforms into the susceptible phases α-Fe, Fe_2_B, and NdH_2_ (Figure 10f). When the pressure is lowered to vacuum, the hydrogen is desorbed and recombination and nucleation of new Nd_2_Fe_14_B grains occur (Figure 10g), which then spread until the entire structure is redistributed to ultrafine Nd_2_Fe_14_B grains and an Nd-rich phase (Figure 10h).^47^ In addition, Sepehri-Amin et al.^61^ reported that the Fe_2_B phase is the only phase that maintains its specific crystallographic orientation during the entire d-HDDR process. Based on their results, highly aligned Fe_2_B grains which can be obtained by 30 kPa of hydrogenation pressure memorize the crystallographic orientation of the initial Nd_2_Fe_14_B phase and transfer it to the recombined Nd_2_Fe_14_B grains.

**Figure 11:**
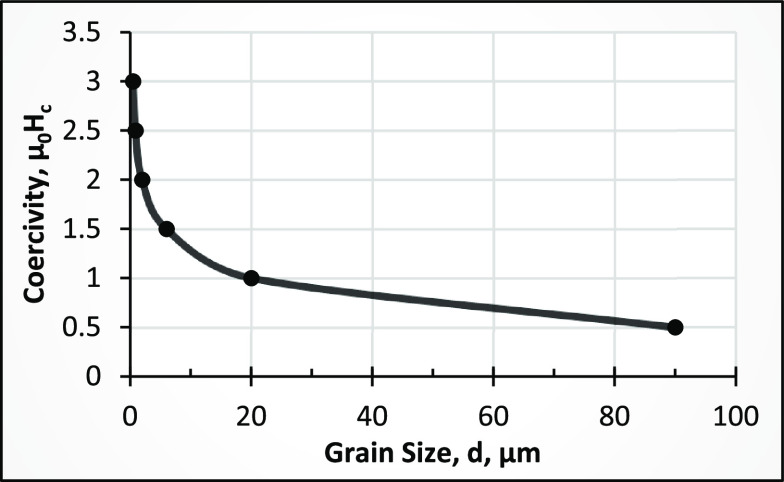
Effect of the grain size on the coercivity of sintered NdFeB magnets. Adapted with permission from ref (46). Copyright 2018 Elsevier.

**Figure 12:**
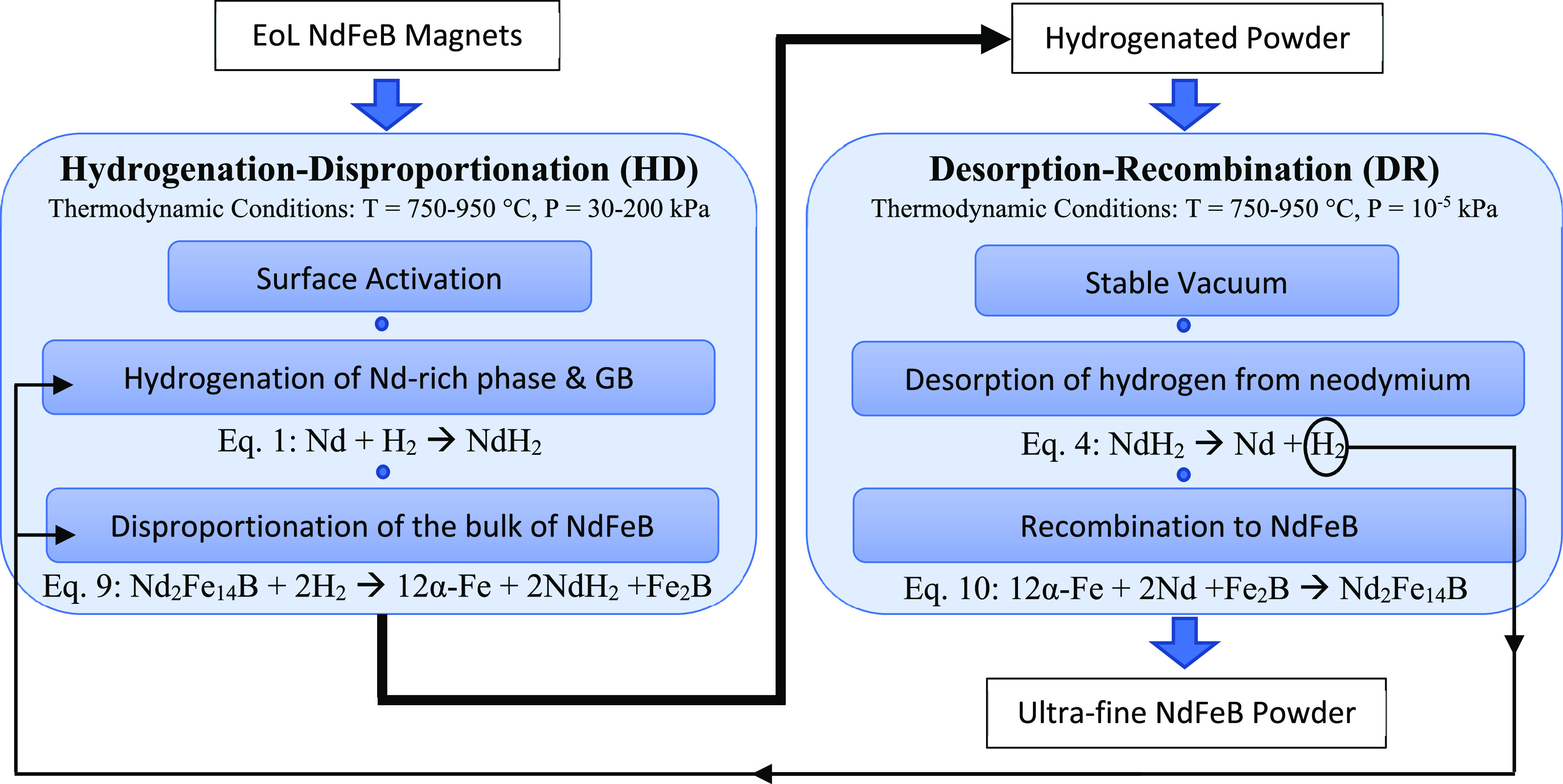
Mechanism flow sheet for recycling an EoL NdFeB magnet through the HDDR process.

The HDDR process is used in the recycling of sintered NdFeB magnets and in the primary production of bonded magnets because it produces coercive powder.^33,46^ The reason for decreasing the grain size by the HDDR process is the magnetic isolation of individual Nd_2_Fe_14_B grains by re-forming a thin continuous Nd-rich phase surrounding the grains. This is one of the mechanisms to increase the coercivity (*H*_cj_), which results in achieving a higher value of the maximum energy product (*BH*)_max_ of the magnets.^62^ The experimental results obtained by Sepehri-Amin^46^ for sintered NdFeB magnets prove that the coercivity decreases with increasing grain size, as shown in Figure 11. The details of the HDDR process for recycling EoL NdFeB magnets including the steps, mechanisms, reactions, and thermodynamic conditions are shown in Figure 12.

**Figure 13:**
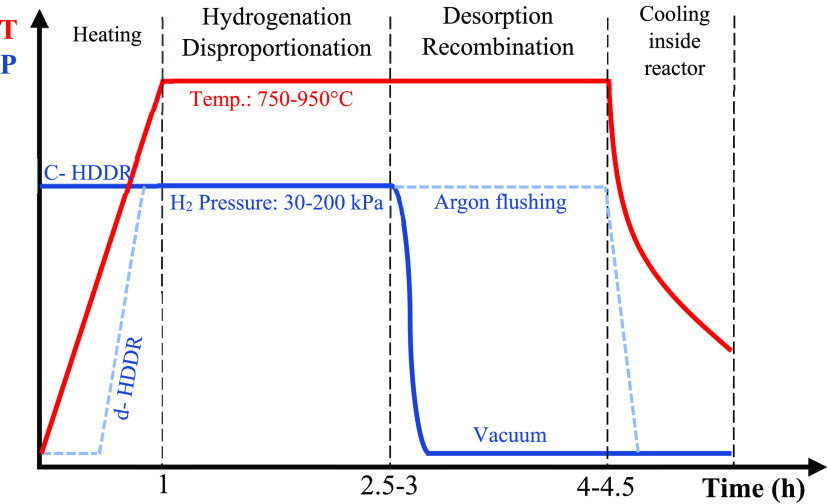
Schematic representation of the HDDR process in terms of time, temperature, and pressure. Adapted with permission from ref (40 and 54). Copyright 2012, 2017 Elsevier.

Despite HD, the HDDR process is carried out continuously. The process includes raising the temperature to a certain value, setting the initial specific pressure that lasts until the vacuum step, and cooling in the reactor after the process is completed. The schematic of the HDDR process in terms of time, temperature, and pressure, taken from the literature, is shown in Figure 13.^40,47,54^

There are two ways to apply the HDDR process. Conventionally, the initial pressure of hydrogen is the operating pressure (blue solid line in Figure 13), and the evacuation of the reactor occurs after the complete disproportionation to start the recombination, which is called conventional HDDR (C-HDDR or HDDR). The other path of the HDDR process starts with a vacuum and then increases to the operating pressure (blue dashed line in Figure 13) before reaching the operating temperature. After complete disproportionation, the reactor is evacuated and filled with argon gas to cause slow recombination and prevent severe supercooling, which is referred to as dynamic HDDR (d-HDDR).^47,54^ In both methods, the temperature starts at room temperature with a specific heating rate to reach a final value and then can be fixed at a specific value or change during processing. The effects of temperature and pressure on the process are discussed in Section 5.

The stoichiometric coefficients of the reactions have been simplified in Figure 12, and the exact coefficients depend on temperature and pressure. Based on the various studies, the more accurate reactions for HDDR are presented as eqs 5, 11, 8, 4, and 10, respectively.^47^
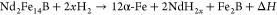
11

**Figure 14:**
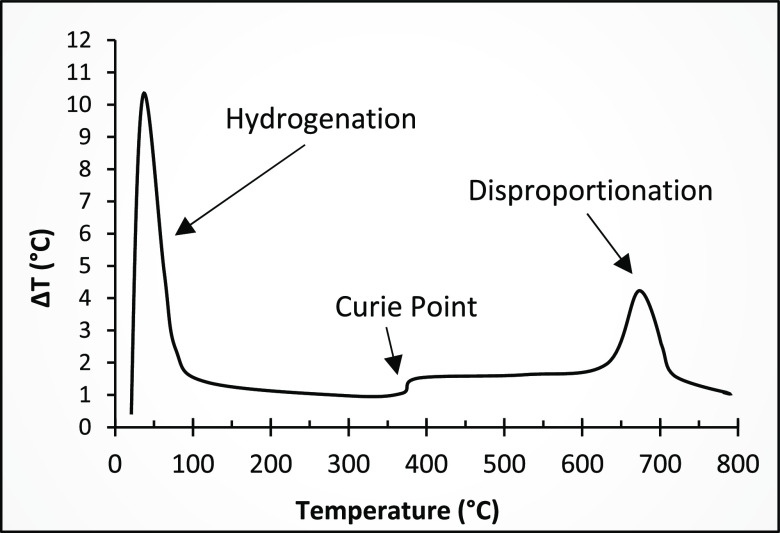
DTA during the HDDR process of NdFeB magnets. Adapted with permission from ref (40). Copyright 2017 Elsevier.

Since eqs 5 and 11 are exothermic reactions, differential thermal analysis (DTA) can show the temperature range for hydrogenation and disproportionation due to the heat released, as shown in Figure 14.^33,40^

## 5 Hydrogen Processing Parameters

The most important challenge in magnet-to-magnet recycling of NdFeB magnets by hydrogenation is the recovery rate of the magnetic properties of the final products rather than the yield weight, since the efficiency of the process can easily reach over 90% if clean, unoxidized, and disassembled waste magnets are provided. One of the main reasons that the hydrogenation process is not widely used is its complexity and the many variables that affect the final magnetic properties. Therefore, all efforts to use HPMS are focused on optimizing process parameters such as temperature and pressure to achieve the highest recovery of magnetic properties.

This section explains all effective parameters and the product properties resulting from these parameters in detail. Table 1 shows the research that has been carried out in the field of HPMS.

**Table 1:** Different HPMS Proposed to Optimize Magnetic Properties Recovery

HPMS type	scrap type	focus of the research	magnetic properties and its recovery rate	ref
HD	HDDs	finding the optimum temperature for hydrogenation and dehydrogenation, best route for degassing, optimizing the entire process	85% (*BH*)_max_ = 290 kJ m^–3^	(53)
HD	HDDs	maintain the magnetic properties and density after multiple recycling (four-cycle)	>90% if blended with 1 wt % fresh powder	(44)
d-HDDR (HD assist)	scrap sintered magnet (NA)	finding the optimum temperature for hydrogenation (835–930 °C) and optimum pressure	(*BH*)_max_ = 129 kJ m^–3^	(54)
HD	scrap sintered magnet (NA)	finding the optimum pressure and temperature of the process, measuring the hydrogen and oxygen content	(*BH*)_max_ = 56.3 kJ m^–3^	(48)
HD	scrap sintered magnet (NA)	comparison of regenerating new magnets via manual crushing (MC) or hydrogen decrepitation (HD) in terms of magnetic properties	HD: (*BH*)_max_ = 111.6 kJ m^–3^	(63)
			MC: (*BH*)_max_ = 91.4 kJ m^–3^	
HD	HDDs	introducing a proto-type separation apparatus and scaling up to the industrial level, measuring the magnetic properties after producing new magnets by sintering	>90% by resintering route	(28)
d-HDDR	HDDs	microstructural study of NdFeB magnets during each step of HDDR process	NA	(47)
HDDR (HD assist)	electric motor, HDD loudspeaker	study the effect of pressure, temperature, and initial chemical composition on the magnetic properties	∼90%	(40)
HD, HDDR	voice coil magnet (VCM), low grade (LG) high grade (HG)	study several hydrogenation treatments including HD, vHD, cHD, PD, FD, and thermal oxidation stabilities, study their comparative oxidation behavior and microstructural changes	NA	(59)
HD	commercial grade 42H of NdFeB magnet	study the effect of H_2_ pressure on the process of sintered NdFeB strip casting flakes (SC) and waste sintered magnets (SM), effect of pressure on surface activation	NA	(51)
HD	commercial cuboids NdFeB N42	determining the optimal temperature and pressure of the process	NA	(49)
HD	HDDs	finding the optimum temperature and pressure of the process and their effect on the magnetic properties	(*BH*)_max_ = 121 kJ m^–3^	(52)

Experimentally, HPMS is a function of initial chemical composition (icc), temperature (*T*), and pressure (*P*). The product of this function is the magnetic properties (*BH*)_max_ with a specific coefficient (θ). This coefficient depends on the particle size (PS), grain size (GS), and oxygen content (OC) with their coefficients (*a*, *b*, *c*), as can be presented briefly in eq 12.
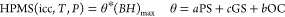
12

It should be noted that the effects of temperature and pressure on the hydrogenation process interact with each other.

### 5.1 Effect of Hydrogenation and Dehydrogenation Temperatures

In hydrogenation, the major difference between HD and HDDR is the operating temperature, which leads to decomposition at lower temperatures (25–400 °C) for HD and disproportionation at higher temperatures (750–950 °C) for HDDR. However, the variations of temperature in both HD and HDDR and dehydrogenation have some effects on the properties of the final products, which will be explained in this section based on the literature.

#### 5.1.1 Hydrogen Decrepitation and Dehydrogenation

Zakotnik et al.^44,53^ reported that the solubility of hydrogen in the Nd_2_Fe_14_B compound decreases with increasing temperature. Therefore, at a higher temperature, a lower amount of hydrogen is absorbed which is not sufficient for fracture but allows the hydrogen atoms to diffuse to a greater depth before fracture, resulting in the formation of large particles. They found that the powder obtained by hydrogenation at 25 and 150 °C has the largest fraction with sizes of less than 250 and 500 μm, respectively. At the same time, hydrogenation at 300 and 450 °C results in more coarse fractions. According to this report, 150 °C is the optimum temperature for hydrogen decrepitation in terms of magnetic properties. As for the optimum dehydrogenation temperature, the intrinsic coercivity (*H*_cj_) and remanence (*B*_r_) were measured at different dehydrogenation temperatures (200–1000 °C). The observations of Zakotnik et al.^44,53^ confirmed that dehydrogenation at a temperature higher than 700 °C leads to a decrease in magnetic properties. They attributed this phenomenon to grain growth. In addition, Piotrowicz et al.^49^ reported that the maximum hydrogen uptake into the magnet’s bulk decreases significantly with increasing hydrogenation temperature. This group of researchers stated that the temperature should be less than 100 °C to achieve complete decrepitation and that samples subjected to hydrogenation at higher temperatures partially decrepitated, and the lowest degree of decrepitation occurs at 400 °C.

The amount of oxygen absorbed during hydrogenation and dehydrogenation is an important factor influencing the final magnetic properties. The presence of oxygen can cause the formation of neodymium oxide (mainly Nd_2_O_3_) at the grain boundary and decrease the degree of magnetic isolation of individual Nd_2_Fe_14_B grains, leading to a decrease in coercivity. In addition, the formation of neodymium oxide decreases the Nd content, which may contribute to liquid sintering. Only the NdFeB magnet can achieve a value of over 400 kJ/m^3^ for the (*BH*)_max_, which is only achieved by using a low-oxygen process.^46^ Lixandru et al.^40^ proved that oxygen content always increases after hydrogenation. Li et al.^48^ measured the oxygen content of hydrogenated powder at different temperatures. They found that the minimum oxygen content belonged to the sample hydrogenated at 150 °C. Furthermore, according to their report, 600 °C is an efficient temperature for removing hydrogen from the system.

A recent study by Michalski et al.^52^ showed that the effects of temperature and pressure on the process are complex and interrelated. First, they reported that using higher temperatures for decrepitation dramatically reduces the initialization time and duration of the hydrogenation process. It was also observed by them that as the temperature increases, the total volume of absorbed hydrogen decreases, but the trend is not the same at different operating pressures. The experiments were conducted at four different temperatures: 50, 100, 200, and 300 °C. According to their results, the size fraction 100–160 μm formed more at 200 °C of hydrogenation. The authors concluded that the choice of the optimum temperature depends on the operating pressure and indicated 50 °C at 50 kPa as the optimum process condition for hydrogenation. For dehydrogenation, they tested a range of 720–820 °C and preferred 780 °C as the optimum dehydrogenation temperature. They also introduced the hybrid process, a continuous process in which the powder is not removed from the reactor after hydrogenation but vacuum and dehydrogenation are started to reduce oxidation in one pass. In this process, the operating temperature for hydrogenation is 200 °C at an initial pressure of 200 kPa, which then decreases to 50 kPa, and 780 °C is set as the dehydrogenation temperature in the final stage.

#### 5.1.2 Hydrogenation–Disproportionation–Desorption–Recombination

**Figure 15:**
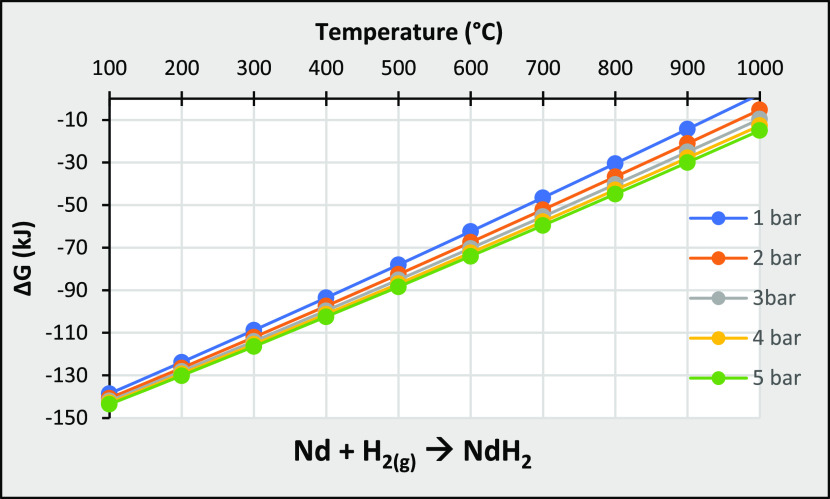
Gibbs free energy of neodymium(II) hydride formation in different temperatures and pressure. Extracted from Factsage software.

**Figure 16:**
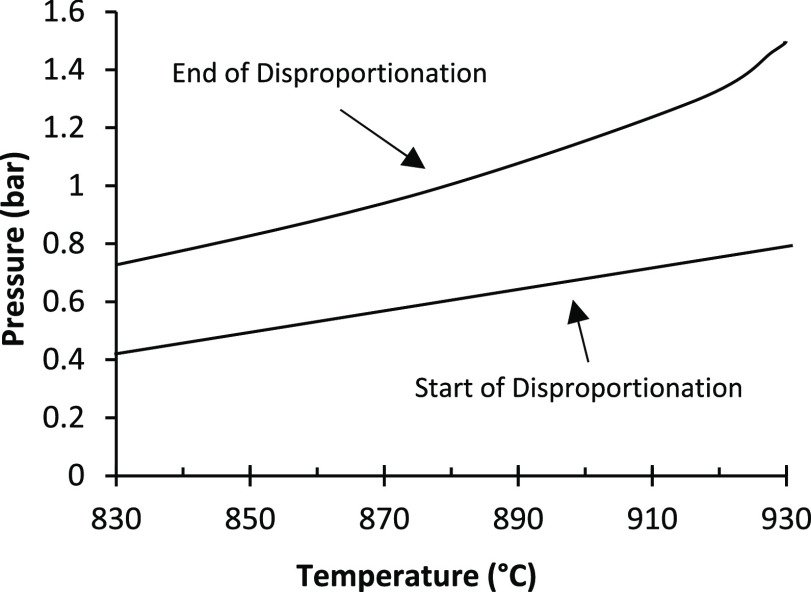
Starting and ending points of disproportionation in different pressure and temperature. Adapted with permission from ref (54). Copyright 2012 Elsevier.

As mentioned above, the minimum temperature for disproportionation of the Nd_2_Fe_14_B compound is 650 °C, since the α-Fe phase is unstable below this value. The interaction of temperature and pressure effects on the HDDR process has been well explained by Sheridan et al.^54^ The Gibbs free energy of neodymium hydride formation increases with increasing temperature and decreases with increasing pressure, as shown in Figure 15 (data from Factsage software). Thus, with increasing temperature, eq 11 reverses, while with increasing pressure it proceeds to the products. They also measured and estimated the beginning and end of disproportionation at different temperatures and pressures, as shown in Figure 16, implying that each pressure has a different optimum temperature. Magnetic properties were also measured by these researchers at different disproportionation temperatures (835–930 °C). They recorded the highest value of intrinsic coercivity (*H*_cj_) and remanence (*B*_r_) at 880 °C for 1 bar hydrogen pressure during disproportionation.^54^ Since HDDR is a continuous process, the disproportionation temperature is maintained while the pressure simultaneously decreases to the vacuum level to provide the necessary conditions until dehydrogenation and recombination (DR) are completed.

Lixandru et al.^40^ proposed a new process consisting of a two-step temperature treatment for the hydrogenation process to achieve maximum recovery of magnetic properties from scrap magnets with a higher weight fraction of Dy and Co. They also reported that higher temperatures of dehydrogenation (around 900 °C) lead to a decrease in magnetic properties due to grain growth. Based on their experimental results in a range of 680–890 °C for the operating hydrogenation temperature, they kept their samples at 780 °C for 30 min, and then the reactor was heated to 840 °C and kept for 3 h until the end of the process as the optimum condition.

### 5.2 Effect of Hydrogenation Pressure

As explained in the previous section, the optimum pressure varies with temperature: i.e., each process at a given temperature has its optimum pressure. However, a different pressure may affect the process regardless of the interaction with temperature. The pressure range studied varies between 0.5 and 15000 kPa for the HD process and between 30 and 200 kPa for the HDDR process. Li et al.^48^ studied hydrogenation at room temperature and 1–3 bar pressure. They explained that higher pressure can lead to intense hydrogenation reactions with enormous amounts of heat, resulting in more oxidation of the powder.

Li et al.^51^ conducted and reported the results of their study on the effects of a wide pressure range (0.09–15 MPa) on the hydrogenation process. First, they measured the time for initialization of hydrogenation (surface activation) at different pressures. They found that the time required to start hydrogen decrepitation decreases from 3000 to 300 s when the pressure is increased from 0.09 to 1 MPa. At pressures higher than 6 MPa, surface activation and decrepitation begin immediately after hydrogen is injected. Second, the amount of hydrogen absorbed depends not on the pressure, but the chemical composition; more rare-earth elements in the magnets lead to higher hydrogen content. Third, the sieved hydrogenated powder results in three ranges of particle size, including 0–200, 200–450, and larger than 450 μm. As the pressure increases from 6 to 15 MPa, the 0–200 μm size fraction decreases, while the faction of particles larger than 450 μm increases. Finally, they found that the overall hydrogenation process accelerates, and the blasting power reduces with increasing pressure. Despite the results found by Li,^51^ Piotrowicz et al.^49^ reported a slight increase in maximum hydrogen uptake by increasing the pressure from 2 to 4 bar. It was reported that complete decomposition occurs at an operating pressure of 2–4 bar.

Michalski et al.^52^ studied two different pressures (50 and 200 kPa), hydrogen flow rates, and the related effects on the hydrogen decrepitation process. The equipment they used allowed instantaneous monitoring of the pressurized hydrogen flow in the reactor. In agreement with Li,^51^ Michalski et al.^52^ also reported a significant reduction in the initialization time of the HD process by increasing the pressure from 50 to 200 kPa. By monitoring the hydrogen flux, they found that hydrogen absorption at higher pressure (200 kPa) frequently fluctuates at the beginning and becomes more stable as the process progresses. In comparison, at lower pressure (50 kPa), it is steady from the beginning and spreads out over a more extended period. The effect of pressure on the particle size of 100–160 μm was not clear due to the interaction with the temperature effect. At lower temperatures (50, 100 °C), increasing pressure causes a reduction of particle size, while at higher temperatures (200, 300 °C) it has the opposite effect. The magnetic properties of all samples at any temperature are higher at low pressure. Finally, they declare that the optimal pressure for a more sustainable process is 50 kPa.

Lixandru et al.^40^ studied the effects of changing the pressure in a range from 30 to 110 kPa and the desorption rate in a range from 0.3 to 45 L/min on the HDDR process. The XRD results showed that the hydrogen pressure in the range of 30–50 kPa was not sufficient for complete disproportionation. In terms of magnetic properties, improved properties were observed by increasing the pressure by more than 30 kPa up to an upper limit of 110 kPa and a desorption rate of about 15 L/min. A very low desorption rate (∼0.3 L/min) is not sufficient to complete dehydrogenation in normal time, and a very high desorption rate (>15 L/min) has a detrimental effect on texture due to severe supercooling caused by rapid evacuation, both of which result in lower coercivity.

### 5.3 Effect of Initial Chemical Composition

**Figure 17:**
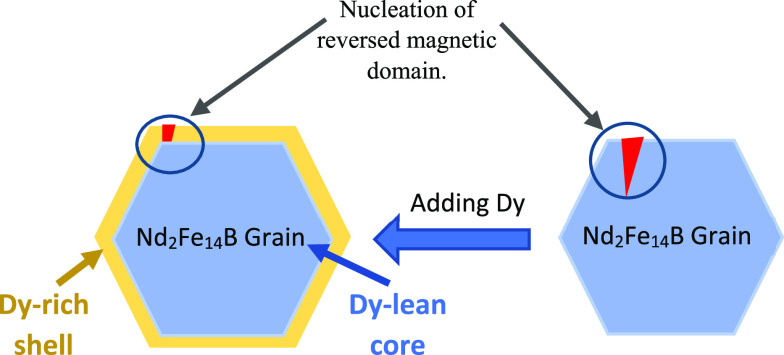
Mechanism of increasing magnetic isolation of Nd_2_Fe_14_B grains by the formation of a core–shell structure. Adapted with permission from ref (39). Copyright 2015 Elsevier.

In the development of NdFeB magnets, different additives (mostly HREEs) have been introduced to the Nd_2_Fe_14_B matrix over the years to enhance the magnetic properties. For example, dysprosium is one of the well-known HREEs added to the NdFeB magnet in the case of application at elevated operating temperatures to increase the Curie temperature (*T*_c_) of the magnet and magnetic isolation of Nd_2_Fe_14_B grains.^31^ The mechanism for increasing demagnetization resistance and magnetic isolation of Nd_2_Fe_14_B grains by adding Dy is the formation of a core–shell structure. Based on this mechanism, the Dy diffuses to the grain boundary and forms a layer around the grain that prevents any nucleation with reversed magnetic domain from further growth, as shown schematically in Figure 17.^39^ In addition, higher anisotropy of the magnetic field was observed in (Nd,Dy)_2_Fe_14_B compared to Nd_2_Fe_14_B, which is another result of the formation of a core–shell structure.^64^ Zhou et al.^65^ reported the formation of a double-layer core–shell structure by adding Tb and Dy in the form of (Nd,Dy)_2_Fe_14_B and (Nd,Dy,Tb)_2_Fe_14_B by injecting TbH_2_ into magnets. In another study by Sepehri-Amin et al.,^66^ copper was added to enhance the formation of the Nd-rich phase. As result, they observed the copper segregation to NdO_*x*_/Nd_2_Fe_14_B interfaces, which causes more magnetic isolation of Nd_2_Fe_14_B grains.

Improving the magnetic properties of NdFeB magnets by a core–shell structure in primary production interferes with the hydrogenation process for recycling. The hydrogenation process is based on a gas–solid reaction, which means that the optimal process conditions depend on the equilibrium pressure between the phases, which is a function of the chemical composition.^46^ Furthermore, the presence of Co, Ga, Zr, and Nb alters the kinetics of the hydrogenation process.^54^

Lixandru et al.^40^ observed a shift of the Curie temperature to a higher value in the case of the presence of cobalt in the magnets. They also reported that for magnets with a high weight fraction of Co and Dy, the recovery of coercivity ranges from 37 to 72%. In comparison, small amounts of these two elements lead to a recovery of magnetic properties of about 100%. Therefore, the HPMS process depends on the chemical composition of the magnetic scrap. The optimal temperature and pressure of the process may change with a different type of scrap. This complexity makes the use of the HMPS challenging due to the diversity of the scrap and chemical composition.

The same as the primary production of NdFeB magnets, the key to the recovery of magnetic properties of recycled NdFeB magnets is rooted in doping with REE hydrides.^67^ Liu et al.^68^ reported that by adding up to 1% of nanoparticles DyH_3_ before sintering about 89% of the initial (*BH*)_max_ can be recovered. This technique, which is called grain boundary modification (GBM), modifies the boundary between the REE-rich phase and Nd_2_Fe_14_B matrix grains, leading to the enhancement of magnetic isolation of the grains by rebuilding the core–shell structure.^69^

#### 5.3.1 Effect of Coating

Due to technological challenges in the production procedure of NdFeB magnets and their multiphase structure, there is a high risk of corrosion in a humid environment.^70^ To prevent oxidation and increase the corrosion resistance of NdFeB magnets, they are commonly coated with nickel or Ni-Cu-Ni. Also, there are various other alternatives for coating such as a Ni-Ni(S)-Ni(P) multilayer coating, alumina ceramic coatings, a multilayer titanium nitride ceramic coating, etc.^71−73^ The presence of the coating layer harms the efficiency of the hydrogenation in the recycling process due to protecting the grain boundaries from diffusion and preserving the magnetic properties of NdFeB magnets.^74^ Therefore, the coating layer should be removed partially or completely to allow the hydrogen gas to enter the magnet. Otherwise, a higher initial pressure of hydrogen is required to start the hydrogenation process.^51^ The common nickel coating with a thickness of about 13 μm can be easily removed or damaged manually or by electrochemical technology.^28,49,53,74^ Damaging the coating by bending and breaking the magnets is preferable to complete decoating, as this provides sufficient free surface area for hydrogen diffusion and prevents a higher degree of oxidation.^28^ Piotrowicz et al.^49^ reported that the optimal pressure of hydrogenation decreases from 2–4 bar to 1–2 bar by removing the coating layer before hydrogenation. Michalski et al.^52^ reported that some damage occurred to EoL NdFeB magnets collected from hard drives during magnet separation without intentional intervention. This degree of damage to the coating is sufficient to initiate hydrogenation at low temperatures. After hydrogenation, the coating layer remains as a coarse fraction up to a few centimeters long and can be easily separated by suitable sieving and even with the hands, since it does not interact with the hydrogen gas.^28,52^

## 6 Characteristics of HPMS Products

The product of the hydrogenation process is a hydride-containing powder, which can be a decrepitated powder (HD) or a disproportionated powder (HDDR), depending on the processes described. After dehydrogenation in both processes, the product is a fine NdFeB powder with different particle sizes. In this section, the particle size distribution is briefly explained.

**Figure 18:**
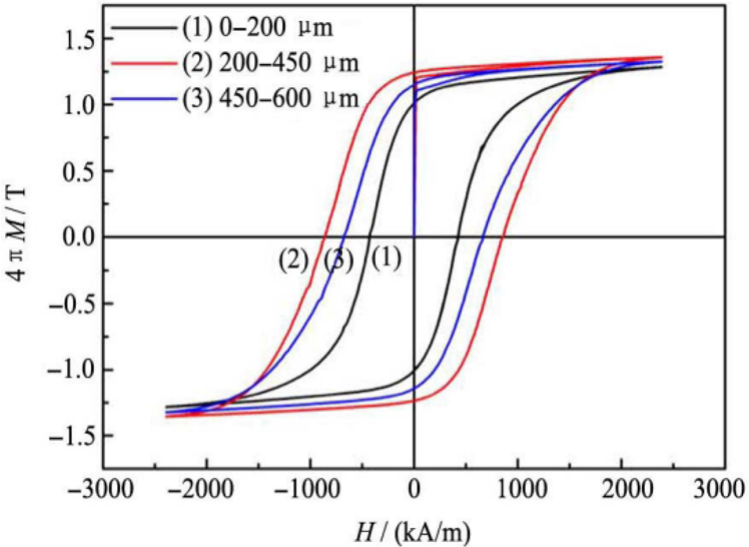
Effect of particle sizes of recycled NdFeB magnet powder by hydrogenation on magnetic hysteresis loop. Reprinted with permission from ref (63). Copyright 2015 Elsevier.

One of the most important features of HPMS is the particle and grain size distribution of the powder. The importance of particle size distribution is related to the oxygen content of the particles, which results in direct effects on the final magnetic properties. As described by Li et al.,^48^ the oxygen content increases as the size of the powder particles decreases due to a larger specific surface area. The oxygen content of particles below 50 μm is almost twice that of particles above 150 μm. As mentioned earlier, higher oxygen concentrations lead to the deterioration of magnetic properties due to the formation of neodymium oxide at the grain boundaries. Based on their measurements of remanence and coercivity, the highest value of the maximum energy product belongs to particles above 380 μm with (*BH*)_max_ = 166.4 kJ/m^3^, while it decreases to (*BH*)_max_ = 56.3 kJ/m^3^ for a magnet made of all particle sizes.^48^ Zakotnik et al.^53^ also measured the coercivity in particles of different sizes and found that the lowest value of the intrinsic coercivity belongs to particles below 75 μm. By extending Li’s^63^ experiments, it was found that even very large particles (>450 μm) can cause a drop in magnetic properties. Li et al.^63^ divided the NdFeB powder they prepared into three size categories (0–200, 200–450, and 45–600 μm), measured the magnetic properties separately, and drew the intrinsic hysteresis loop for them as shown in Figure 18. A particle with a size of 200–450 μm has the highest maximum energy product because the largest rectangle that can be fitted into the second quadrant of hysteresis loop is larger compared to other particle sizes. The better magnetic properties of the medium-sized particles (200–450 μm) compared to the large-sized particles (450–600 μm) are attributed to the fact that the medium-sized particles contain fewer grains and therefore have better crystallographic alignment.^63^

The particle size distribution was determined by Michalski et al.^52^ after a hydrogenation process at 200 °C and 200 kPa for temperature and pressure, respectively, and dehydrogenation at 780 °C, as shown in Table 2. These data show that the final magnetic properties will be more similar to those of the fine fractions with a particle size below 200 μm due to their weight percentage. In addition, the best magnetic properties were determined for the coarse fraction with a particle size of 400–500 μm.

**Table 2:** Particle Size Distribution for Powder Processed at 200 °C and 200 kPa^a^

particle size (μm)	fraction weight (%)
0–200	51
200–400	28
400–700	21

a Adapted with permission from ref (52). Copyright 2022 Elsevier.

## 7 Conclusions

Recycling NdFeB magnets from secondary sources is one of the promising ways to overcome the supply risk of critical raw materials such as Nd and Dy. HPMS as the shortest way to recycle EoL magnets with high efficiency has been detailed in this review by describing the hydrogen decrepitation (HD) and hydrogenation–disproportionation–desorption–recombination (HDDR) as two common routes to hydrogen processing. The main challenge in this recycling method is the fact that the recycling efficiency mostly depends on the recovery rate of the final magnetic properties rather than the efficiency of the process. The recovery rate of the magnetic properties depends on influencing parameters such as pressure, temperature, oxygen content, initial chemical composition, and additives. Another challenge is the need for systematic waste collection to obtain clean, unoxidized, and disassembled NdFeB magnets.

Increasing the hydrogenation temperature in the HD and HDDR processes leads to a decrease in the initialization and duration time of the processes. In the HD process, the particle size of the powder increases by increasing the temperature in the range of 25–400 °C, leading to lower oxygen content and a higher coercivity recovery rate. In the HDDR process, increasing the temperature in the range of 750–950 °C causes grain growth and consequently a drop in (*BH*)_max_.

Overall, the final magnetic properties of the NdFeB magnet decrease with increasing pressure above 1 bar (100 kPa) in both HD and HDDR processes. Other results of a pressure increase in HD and HDDR processes include acceleration of the entire process, decrease in surface activation time, and a slight increase in absorbed hydrogen, resulting in complete decrepitation or disproportionation and an increase in oxidation. The effect of increasing pressure on the particle size of the hydrated powder is not clear, causing a decrease at lower temperatures and an increase at higher temperatures.

The hydrogenation process is sensitive to the interplay between temperature and pressure, with each factor directly influencing the other. As a result, an ideal temperature range can be identified for each operating pressure, and vice versa. For the HD process, it is recommended to carry out hydrogenation at 100 °C and 1 bar pressure, while the optimal temperature for dehydrogenation is below 700 °C to avoid undesired grain growth. In the case of the HDDR process, the optimal operating temperature should be kept below 900 °C.

While the presence of additive elements such as Co and Dy has a terminating effect on the restoration of magnetic properties after the hydrogenation process, this effect can be neutralized up to 90% by adding REE hydrides such as DyH_3_ up to 1% or fresh powder of NdFeB up to 5% before sintering to induce the re-formation of a core–shell structure.
